# High-Selectivity Bandpass Filter with Controllable Attenuation Based on Graphene Nanoplates

**DOI:** 10.3390/ma15051694

**Published:** 2022-02-24

**Authors:** Jianzhong Chen, Jiali Zhang, Yutong Zhao, Liang Li, Tao Su, Chi Fan, Bian Wu

**Affiliations:** National Key Laboratory of Antennas and Microwave Technology, Xidian University, Xi’an 710071, China; jianzhong.chen@xidian.edu.cn (J.C.); jializhang98@126.com (J.Z.); flflymanllm@aliyun.com (L.L.); taosu@mail.xidian.edu.cn (T.S.); 18021110202@stu.xidian (C.F.); bwu@mail.xidian.edu.cn (B.W.)

**Keywords:** tunable attenuation, graphene nanoplate, bandpass filter

## Abstract

A high-selectivity band pass filter with controllable attenuation based on graphene nanoplates is proposed in this paper. Graphene with controllable resistance has a good uniform attenuation effect to electric field intensity. The filter utilizes quarter wavelength stepped impedance resonators and mixed electromagnetic coupling to have compact circuits and high performance. The graphene nanoplates are loaded on the microstrip resonator to reduce the electric field intensity, which results in a flat attenuation in the passband. In addition, the filter has two transmission zeros, which lead to a strong selectivity. Finally, a high-selectivity bandpass filter with controllable attenuation is formed. By changing the bias voltage of graphene, a controllable attenuation of 1.64–11.13 dB can be achieved in the working passband centered at 1.36 GHz. In order to validate the concept, the prototype is fabricated and measured. The measurement results are in good agreement with the simulation results. The proposed high-selectivity bandpass filter with controllable attenuation based on graphene nanoplates has widely potential in reconfigurable wireless communication systems and radar systems due to its high integration and versatility.

## 1. Introduction

**Figure 1:**
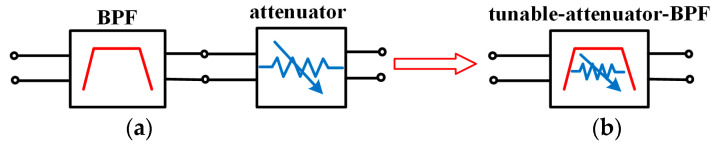
(**a**) Cascaded bandpass filter (BPF) and tunable attenuator. (**b**) Dual-function of filtering and attenuating.

Graphene has attracted much attention due to its light weight, large surface area, excellent optical properties, and electrical properties. In recent years, significant advances have been made in the preparation methods of large-scale graphene [1,2,3], promoting its applications in the microwave and low terahertz ranges [4,5,6,7,8,9], such as RF graphene field effect transistor, graphene antenna, and graphene microstrip attenuator. Graphene nanoplates are composed of few-layer graphene and generally have a small area, which often appear in the form of fragments, suspensions, or powders. Graphene nanoplates provide a new solution for microwave devices. Based on the resistance characteristics of graphene, graphene is introduced in [10] to conduct resistance behavior experiments of coating elasticity. Due to the excellent electrical and optical properties of graphene, a single-layer pure graphene photodetector with a multi-channel parallel electrode structure has been made [11]. Using tunable resistivity characteristics, graphene has been widely used in many microwave devices. For example, antennas [12], attenuators [13,14,15], filters [16,17], and two tunable microstrip filters based on graphene nanoplates are proposed. In [18], graphene is located in the middle of the microstrip line, which describes a method of combining graphene with the microstrip line. In [19], graphene replaces metal plates to implement microwave low-pass filters. With the development of modern microwave devices, microwave devices are more and more widely used and flexible. Device versatility and high integration are becoming increasingly important. Multifunctional devices can effectively reduce circuit size and enhance application domain. As shown in Figure 1a, filters and attenuators are generally used in conjunction, resulting in larger circuit size and poorer performance. The filter and attenuator are merged into one device in Figure 1b, which not only reduces the circuit size, but also improves the performance of the device. Although studies on tunable filtering attenuators are carried out in [16,17], their devices did not have good selectivity. 

In this paper, a high-selectivity bandpass filter with controllable attenuation based on graphene nanoplates is proposed, which can realize the function of tunable passband attenuation. First, an electromagnetic coupling high-selectivity filter composed of λ/4 step impedance resonators is designed [20]. Then, the graphene nanoplates are loaded around the resonant cavity to analyze the influence of graphene on the electromagnetic field distribution. A suitable loading position can be found for graphene nanoplates. The resistance of graphene nanoplates can alter dynamically when different bias voltages are applied to graphene. In this way, the attenuation of the filter passband can be adjusted. Therefore, a high-selectivity bandpass filter with controllable attenuation based on graphene nanoplates can be designed. Finally, a prototype is fabricated and measured to validate the concept. The measured results are in good agreement with the simulation results.

## 2. Materials and Methods

**Figure 2:**
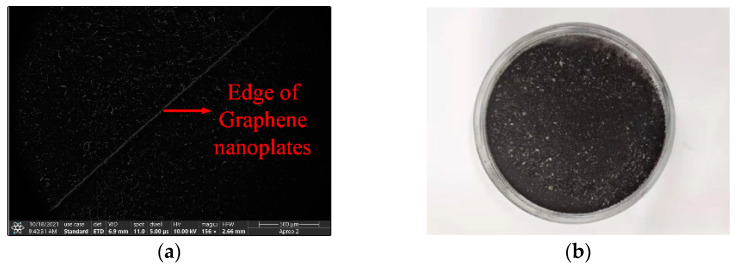
(**a**) SEM image and (**b**) macroscopic physical image of graphene.

The characteristics of graphene nanoplates, such as tunable resistivity, corrosion resistance, high temperature resistance, and ease of fabrication, make them a very important application prospect in microwave devices. In the fabrication process, graphene nanoplate dispersion is prepared at first. The specific fabrication procedure can be described as follows. Graphene nanoplates XF022-1 from a commercial company (XFNANO, Nanjing, China) are mixed with isopropyl alcohol at a concentration of 2.5 mg/mL. The mixture is sonicated for 40 min at 160 W. Graphene nanoplate dispersion is deposited at the specific locations of filter gaps by using a dropper and customized molds. When the isopropyl alcohol solution is completely vaporized, three pairs of graphene pads are formed. The resistance value of graphene nanoplates can be tunable by adjusting the bias voltage. Therefore, when the graphene nanoplates are loaded on the filter, the attenuation of the electric field can be tunable by controlling the bias voltage. Figure 2a is a microscopic image of graphene under Apero 2C scanning electron microscope (SEM). The white line in the picture is the gap where the graphene nanoplates meet the metal plate. Figure 2b is a macroscopic image of graphene. Macroscopically, it is a black powder-like object.

**Figure 3:**
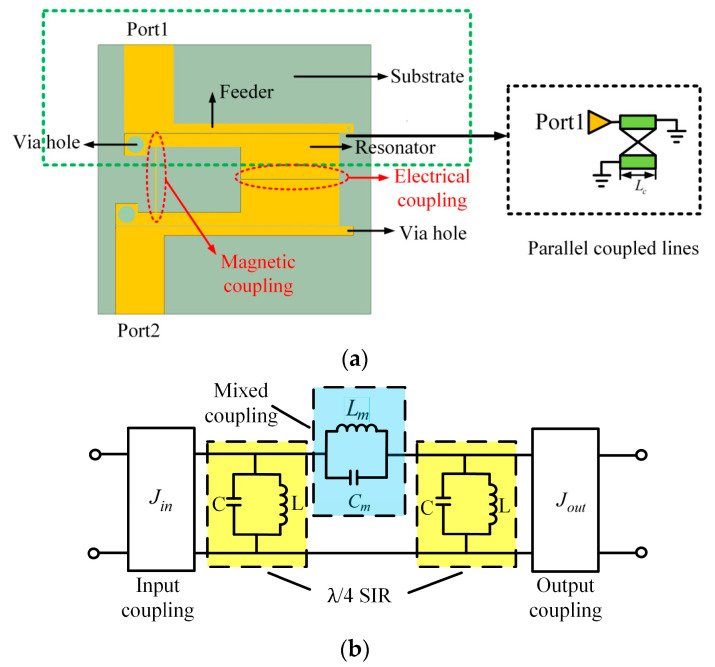
(**a**) Second-order λ/4 stepped impedance resonator (SIR) filter. (**b**) Equivalent circuit.

**Figure 4:**
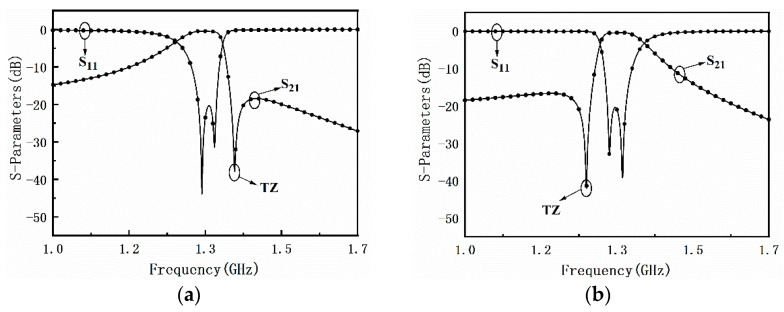
S-parameter simulation results with freely controlled transmission zeros. (**a**) At high frequency stopband. (**b**) At low frequency stopband.

In order to study the effect of graphene nanoplates on the resonator, a second-order filter using λ/4 stepped impedance resonator (SIR) is designed as depicted in Figure 3a. Each resonator has a high impedance and a low impedance region. Graphene nanoplates are loaded on one side of the resonators. The electric coupling between λ/4 SIR is generated between the gap of the coupled low-sections, since the SIR has the maximum electric fringe field density at the open end, which is equivalent to a capacity C_m_ as shown in Figure 3b. On the other hand, the magnetic coupling is achieved by a conducting pin, which taps the two SIRs together and behaves as a coupling inductance and is equivalent to a inductor L_m_ as depicted in Figure 3b. Therefore, the mixed electromagnetic coupling is equivalent to a parallel resonator composed by C_m_ and L_m_. When the mixed electromagnetic coupling structure resonates at a certain frequency, the impedance will be infinity so as to produce a transmission zero (TZ). Because the coupling resonant frequency can be flexibly controlled, the TZs can be located on either side of the filter freely, which can effectively improve the filter’s passband selection characteristics. The simulation results shown in Figure 4a,b confirms the above idea.

**Figure 5:**
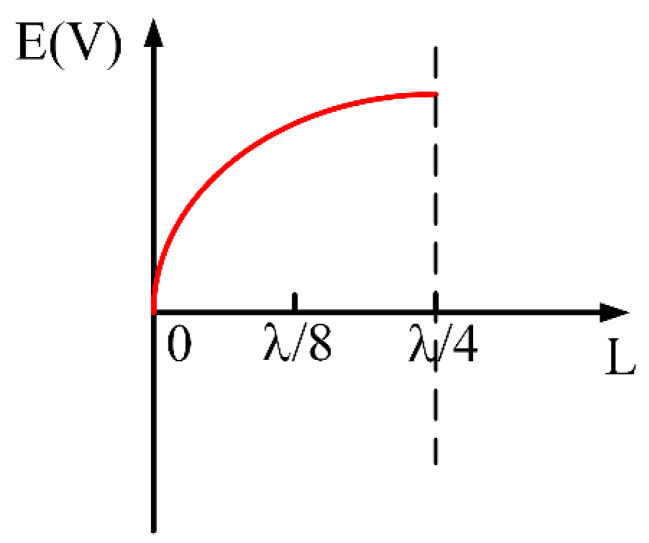
Electric field distribution of λ/4 SIR resonators.

**Figure 6:**
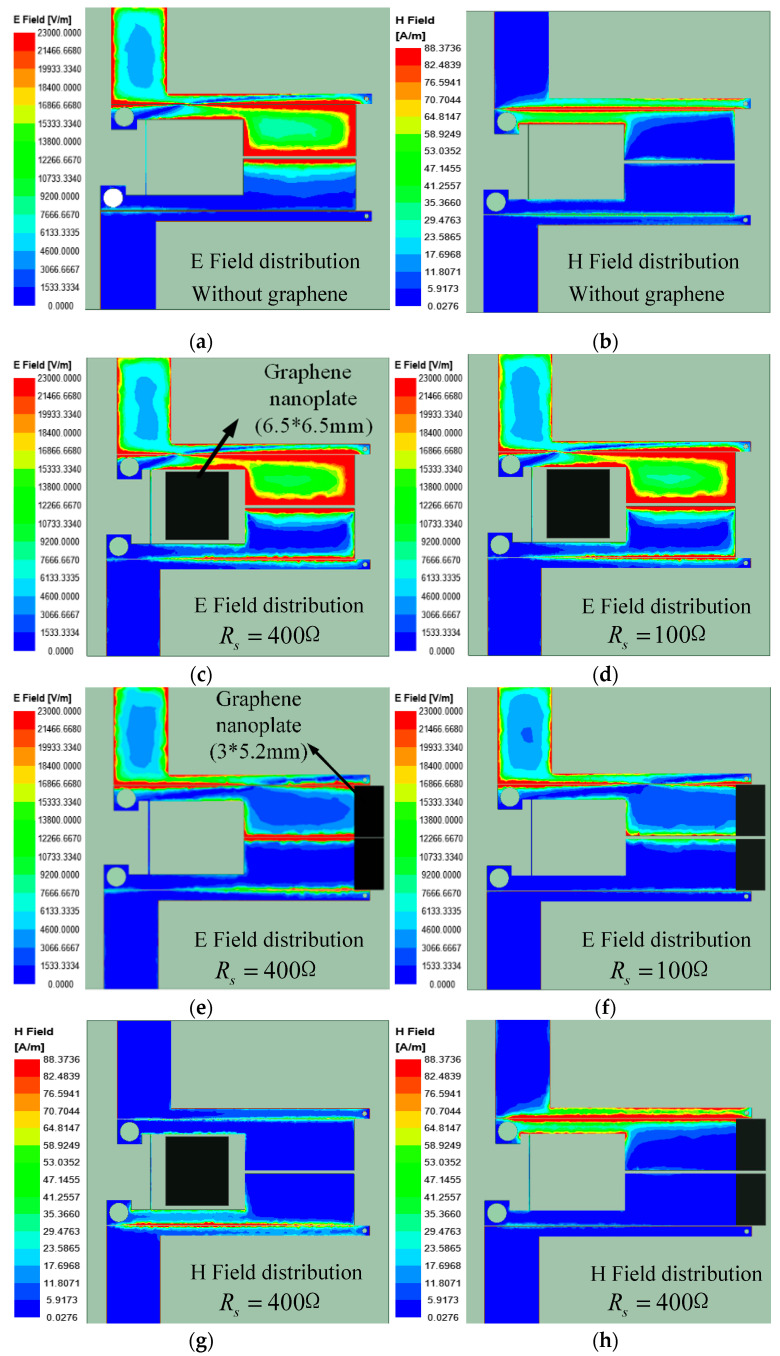
The electric and magnetic field distribution of the filter without (**a**,**b**) and with different graphene nanoplates (**c**–**h**).

Figure 5 shows the electric field distribution of the quarter-wavelength SIR, which can facilitate the analysis of the relationship between loading position and resonance intensity. In order to study the specific effect of graphene on the electric field, the electric field distribution on the filter with grapheme located at different positions is simulated. Figure 6a shows the electric field intensity distribution on the filter resonators. It can be seen that a strong electric field is generated around the filter resonator. Figure 6b shows the magnetic field strength distribution of the filter resonator. Usually, graphene is equivalent to a tunable lumped resistor *R*. The relationship between the simulated value of the sheet resistance $R_{s}$ and the calculated resistance R can be expressed as

(1) $$R_{s}\approx (L/W)\times R$$

where *L* and *W* are the length and width of graphene, respectively.

First, a 400 Ω graphene nanoplate is loaded at a position with a small electric field strength, and the electric field distribution simulation diagram is shown in Figure 6c. Looking at the electric field distribution, the graphene nanoplates has little effect on the electric field. When the resistance value of the graphene nanoplate is changed to 100 Ω, it is found that the influence on the electric field is almost unchanged, as shown in Figure 6d. It is then concluded that adding graphene nanoplates to the filter where the electric field is weak, it has little effect to filter on the attenuation of the amplitude. Figure 6g shows the magnetic field distribution of the model, and it can be seen that loading the graphene nanoplate at the position will affect the magnetic field distribution. It can be seen that adding graphene at this position can attenuate the total energy of the field, but it is not the location that has the greatest effect on the field attenuation. Therefore, the loading position of graphene nanoplate is changed, and the graphene nanoplate is located in the place where the electric field of the resonator is the strongest. Observing the electric field distribution, it can be seen that the electric field around the resonator has a significant attenuation as depicted in Figure 6e. When the resistance value of graphene nanoplates change from 400 to 100 Ω, it can be seen that the graphene nanoplate with different resistance values has different degrees of influence on the electric field. As shown in Figure 6f, the attenuation of graphene nanoplates to the electric field becomes larger. Figure 6h is the magnetic field distribution of the model, it can be observed that graphene nanoplate loading in this position does not change the magnetic field distribution. In summary, the graphene nanoplates should be loaded at the position where the electric field of the resonator is the strongest. Graphene nanoplates have the greatest effect on attenuation of filter energy with this method. When the resistance of graphene nanoplate is reduced, the attenuation of the electric field increases.

**Figure 7:**
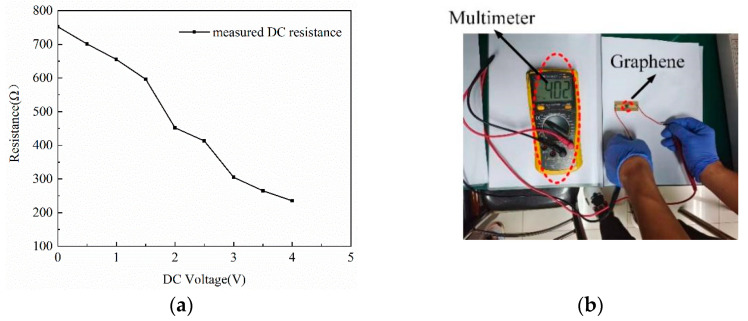
(**a**) Measured DC resistance versus bias voltage. (**b**) Measuring graphene resistance.

Before designing the model, a DC voltage source is used to power up the microstrip line with the graphene. The multimeter measures the DC resistance under bias current, and records the change in resistance as the voltage changes. The relationship between the resistance and the bias voltage is shown in Figure 7. When the bias voltage is 0 V, the resistance is 402 Ω. Figure 7 shows the resistance decreases linearly with increasing voltage. The graphene will generate excessive heat as the DC voltage exceeds 4 V. It will be broken down when the DC voltage reaches about 5 V. By testing the microstrip lines and graphene nanoplates, it is proved that the resistance of graphene can be adjusted, which can control the resonance intensity of the microstrip.

## 3. Experiment Results and Discussion

The above mixed electromagnetic coupling filter only introduces transmission zeros in the high frequency stopband or low frequency stopband. The number of filter stages is also small and the out-of-band drop is slow, which is not suitable for high selectivity scenario. Higher-order filters can provide steeper transition bands to obtain a better square coefficient. In addition, constructing multiple sets of hybrids coupling structure can introduce multiple transmission zeros at the same time, thereby further improving the passband selection characteristics of the filter.

In order to achieve practical application, a third-order SIR high-selectivity filter with tunable attenuation is constructed. Because the transmission zeros of the mixed electromagnetic coupling filter are individually controllable (discussed in Section 2), one transmission zero can be generated on each side of the passband. The center frequency of the filter is $f_{0}=1.36\mathrm{GHz}$, the equal ripple bandwidth BW=80MHz. 

**Figure 8:**
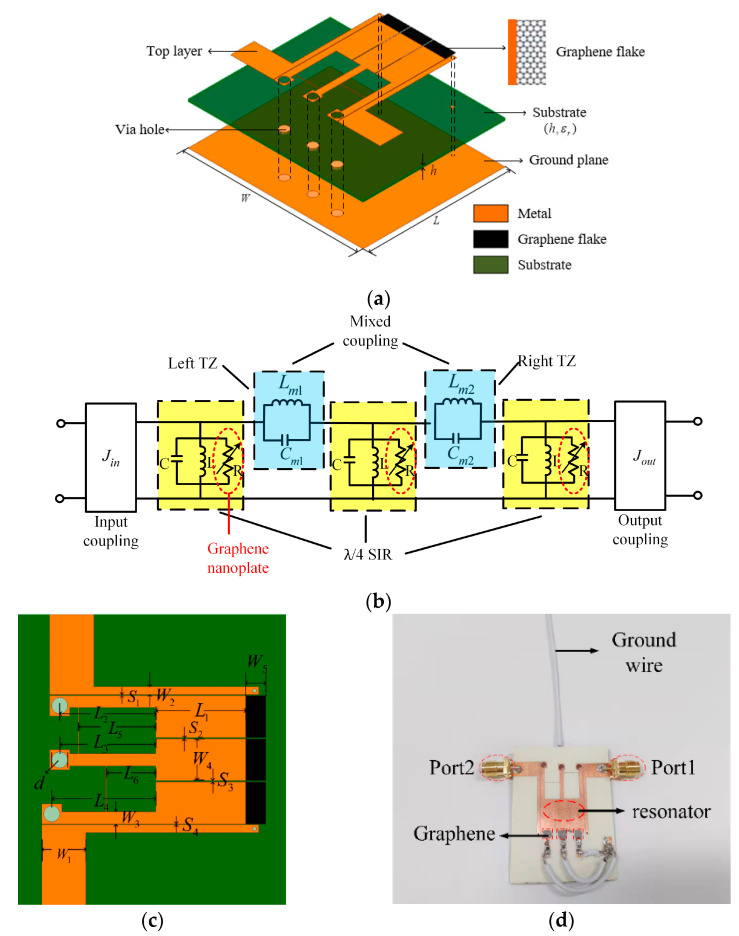
(**a**) Schematic diagram, (**b**) equivalent circuit, (**c**) top view and (**d**) physical view of proposed tunable filtering attenuator (L1 = 11.2 mm, L2 = 12 mm, L3 = 12 mm, L4 = 13 mm, L5 = 9.63 mm, L6 = 6.2 mm, W1 = 5.5 mm, W2 = 1 mm, W3 = 1.5 mm, W4 = 5.2 mm, W5 = 3 mm, S1 = 0.105 mm, S2 = 0.2 mm, S3 = 0.17 mm, S4 = 0.11 mm, D = 2 mm).

The schematic diagram of a tunable resistance graphene-based bandpass filter with mixed electric and magnetic coupling is shown in Figure 8a. It is composed of coupling lines, λ/4 stepped-impedance (SIRs), mixed electromagnetic coupling and graphene nanoplates. The microstrip circuit is made on Rogers 4350 (relative dielectric constant $\varepsilon_{r}=3.66$, loss tangent tanδ = 0.009, h = 0.508 mm). The metal through-hole diameter is d, connecting the upper and lower metal layers with the ground. Three pieces of graphene nanoplate are located at the strongest electric field as tunable resistors. The graphene nanoplates connected in parallel with the resonator cavity will gradually consume the input energy, which can achieve the tunable passband attenuation. Figure 8b is the equivalent circuit of the model to better understand our design. The detailed dimensions of the filter are shown in Figure 8c. Figure 8d is the photograph of the filter based on graphene nanoplates. 

**Figure 9:**
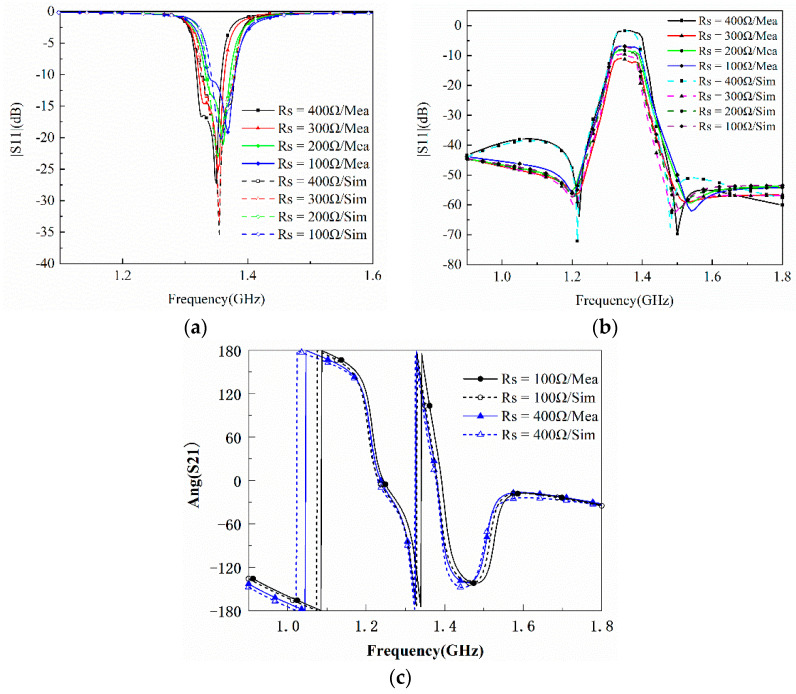
Comparison of the simulated and measured results for the proposed filter with tunable attenuation. (**a**) Magnitude S11. (**b**) Magnitude S21. (**c**) Phase of S21.

The graphene nanoplates are regard as lumped resistors. When the value of R is high, graphene nanoplates are close to open circuit, resulting in small attenuation. By increasing the bias voltage, the value of R decreases and the absorptive of graphene nanoplates become more obvious, which lead to higher attenuation. Figure 9a,b respectively represent the simulated and measured transmission and reflection responses of the filtering attenuator. Under different bias voltages, the attenuation amplitude of S21 can be adjusted to −1.64~−11.13 dB, and the reflection is less than −10 dB. Figure 9c shows the phase responses of S21. It can be found that the phase of the transmission coefficient basically remains unchanged at different attenuation level. In conclusion, it can be seen that the measured results are basically consistent with the simulation results from the Figure 9.

Table 1 gives the comparison among the proposed filter with tunable attenuation and previously reported graphene-based filters/attenuators. It can be found that the proposed filter has the largest passband attenuation range and the best rectangle coefficient, which indicates the best performance. 

## 4. Conclusions

In this paper, a high-selectivity bandpass filter with controllable attenuation based on graphene nanoplates is proposed. Applying different bias voltages to the graphene to maintain low reflection loss, the attenuation amplitude can be adjusted to more than 10 dB. Experiments have proved that controlling the bias voltage on graphene can change the resistance value of graphene, and achieve adjustable attenuation in the resonant structure. The measured results and the simulation results are basically consistent. The proposed tunable resistance graphene-based bandpass filter combines the functions of the filter and the attenuator well. It simplifies the building steps of the radio frequency system and reduces the production cost. The tunable resistance graphene-based bandpass filter design structure is simple and can be well transplanted to the design of other filters. It has very good application prospect in the development of the communication systems.

## Figures and Tables

**Table 1:** Comparison between this design and previous tunable filters based on graphene.

Ref.	|S21| (dB)	|S11| (dB)	Filter Response	Transmission Zeros	Rectangle Coefficient	Structure
[13]	5–10.5	<5	No	No	/	Graphene + MS
[14]	0.3–14	<5	No	No	/	Graphene + MS
[15]	1.7–8.4	<10	Yes	1	4.6	Graphene + HMSIW
[16]	3–15	<15	No	No	/	Graphene + SIW
[19]	2.5–14	<10	No	No	/	Graphene + GCPW
This Work	1.64–11.13	<10	Yes	2	2.12	Graphene + MS

Rectangle coefficient of gain: $K=\Delta f_{-30dB}/\Delta f_{-3dB}$. MS = Microstrip; GCPW = Ground Coplanar Waveguide; HMSIW = Half-Mode Substrate Integrated Waveguide.

## Data Availability

Data sharing is not applicable.
